# Exploring Spatial Overlap of High-Uptake Regions Derived From Dual Tracer Positron Emission Tomography–Computer Tomography Imaging Using ^18^F-Fluorodeoxyglucose and ^18^F-Fluorodeoxythymidine in Nonsmall Cell Lung Cancer Patients: A Prospective Pilot Study

**DOI:** 10.1097/MD.0000000000000678

**Published:** 2015-05-01

**Authors:** Jing Liu, Chengqiang Li, Man Hu, Jie Lu, Xiaorong Shi, Ligang Xing, Xindong Sun, Zheng Fu, Jinming Yu, Xue Meng

**Affiliations:** From the Department of Radiation Oncology and Shandong Province Key Laboratory of Radiation Oncology (JL, CL, MH, JL, XS, LX, XS, JY, XM); PET/CT Center (ZF) Shandong Cancer Hospital and Institute, Shandong University, Jinan, China.

## Abstract

Interest is growing in radiotherapy to nonuniformly boost radioresistant regions within nonsmall cell lung cancer (NSCLC) using molecular imaging techniques. The complexity of tumor behavior is beyond the ability of any single radiotracer to reveal. We hold dual tracer positron emission tomography–computer tomography (PET/CT) imaging with fluorodeoxyglucose (FDG) and fluorodeoxythymidine (FLT) for NSCLC patients to offer an integrated overlook of tumor biological behaviors quantitatively and localizationally, which may help biological target volume delineation and subvolume boost.

Pathological confirmed that NSCLC patients were eligible. FDG and FLT PET/CT were performed for each patient before anticancer treatment and coregistrated for analysis. Maximum and mean standardized uptake values (SUV_max_ and SUV_mean_) were calculated automatically. Metabolic volumes (MVs) were delineated by a fixed 50% of SUV_max_ in FDG PET/CT and proliferative volumes (PVs) were delineated by 50% to 90% of SUV_max_ with 10% interval in FLT PET/CT. Overlap ratio (OR) were determined as overlapped volume between MV and PV divided PV. Conventional contrast-enhanced CT-based intensity-modulated radiotherapy (IMRT) plans with and without additional PET/CT-guided subtarget boost were made for each of the 5 typical NSCLC patients. Dosimetric parameters derived from dose–volume histogram, tumor control probability (TCP), and normal tissue complication probability (NTCP) of lung, esophagus, heart, and spinal cord were calculated and compared.

Thirty-one patients were prospectively included and 23 were selected for analysis. Totally, 23 primary diseases, 41 metastatic lymph nodes, and 15 metastatic lesions were positive in dual PET/CTs and included for analysis. Median ORs increased from 58.61% to 93.12% under thresholds of 50% of SUV_max_ in FDG PET/CT and increased thresholds from 50% to 90% of SUV_max_ in FLT PET/CT. Based on conventional IMRT, additional boost to union of high FDG (determined by 50% SUV_max_) and FLT (determined by 80% SUV_max_) uptake subtargets exhibited higher TCP without significant elevated NTCP of lung, esophagus, spinal cord, and heart.

Dual tracer PET/CT of FDG and FLT is suggested for NSCLC patients to guide tumor target delineation in clinical practice. FDG PET/CT is necessary whereas FLT PET/CT may be optional when guiding tumor target delineation clinically. Additional information from randomized trials is required to validate.

## INTRODUCTION

Radiotherapy, which has been estimated to be applied in 70% of cancer patients,^1^ is adopted by almost all nonsmall cell lung cancer (NSCLC) patients to achieve local disease control and/or clinical symptoms relieve. Standard radiotherapy of NSCLC with 60 to 70 Gy is associated with an local recurrence rate of estimated 50%.^2^ Great efforts have been made to facilitate higher radiation dose under modern radiation techniques based on the clear existed association between total dose, local control, and survival of NSCLC patients.^3,4^ However, the interim analysis of the Radiation Therapy Oncology Group 0617 protocol^5^ showed that the higher radiation dose of 74 Gy could not produce a longer survival time than the standard dose of 60 Gy. Although reported adverse events were similar, the injury of high-dose radiotherapy on normal lungs and heart may lead to more deaths than standard dose.^6^ Under the situation of higher radiation dose not performing better, radiation boost to biological target volumes (BTVs) nonuniformly to improve local control,^7^ may likely bring about new hope for dose escalation to radiation-resistant subregions.

Ideal biological target delineation enables visualization of subvolumes exhibiting heterogeneous biological characteristics of pathophysiological processes, including glucose metabolism, proliferation, hypoxia, epidermal growth factor receptor expression, and choline metabolism, within tumor volume. Positron emission tomography (PET) with single radiotracer, such as typically well-studied fluorodeoxyglucose (FDG),^8^ fluorodeoxythymidine (FLT),^9^ and fluoromisonidazole,^10^ has been investigated as possible dose-painting targets. The fact that overexpression of glucose transporter 1 is consistent with increased cellular proliferation, as well as increased glucose uptake and glycolytic metabolism of tumor cells,^11,12^ laid a solid foundation for the tight linkage of glucose metabolic activity and cell proliferation,^13–15^ which were exhibited by PET tracers of FDG and FLT, respectively. Image presentation of tumor glucose metabolism and cellular proliferative activity provides relevant and complementary information for treatment choices made by radiation oncologists.^16,17^ Nevertheless, multitracer-guided subvolume boost in radiotherapy is urgently in need of research.

For these reasons, we investigated the spatial distribution relationship of regional glucose metabolism and proliferation by comparing FLT and FDG uptake on PET/computer tomography (CT) images to explore the spatial location relation between high-uptake regions determined by baseline dual PET tracers of FDG and FLT in NSCLC patients. Additionally, we planned nonuniform radiation dose boost to high FDG and FLT uptake areas with intensity-modulated radiation therapy (IMRT) as a preclinical study to explore the efficacy and feasibility of BTV boost in NSCLC patients.

## MATERIALS AND METHODS

### Patients

Eligible patients were prospectively recruited from February 2013 until December 2013 in our institute. The inclusion criteria were as follows: histopathological confirmed treatment-naive primary NSCLC; completely recorded with a medical history and physical examination, blood count, serum biochemistry, serum tumor marker determination, contrast-enhanced CT of the thorax and abdomen, and enhanced magnetic resonance scanning of the head and neck at diagnosis at diagnosis; and not indicated to undergo surgery because of advanced disease or comorbidities, as well as patients’ decline. The patients who had poor performance status (≥3), diabetes mellitus under poor control, and human immunodeficiency virus-positive were excluded from this study. Tumor-node-metastasis staging and clinical staging were assessed by the American Joint Committee on Cancer (7th edition). Tumor histology was classified using World Health Organization criteria. All procedures performed in studies were in accordance with the ethical standards of the ethics committees of the institute and with the Helsinki Declaration of 1975, as revised in 2000. This study was approved by the institutional review boards and ethics committees of Shandong Cancer Hospital and Institute. Signed informed consent was obtained in all cases.

### FDG and FLT PET/CT Imaging

FDG and FLT PET/CT were performed on separate and consecutive days. No antitumor treatment was allowed either between or before PET/CT scans. Each patient fasted for at least 8 hours before PET/CT scanning. Blood glucose level recorded prior to scanning was in the normal range. Patients were injected intravenously with FDG or FLT, rested in a quiet room for 1 hour, and then an FDG or FLT PET/CT scan was acquired on an integrated PET/CT scanner (GEMINI TF Big Bore PET/CT; Philips, Amsterdam, The Netherlands). Oral or intravenous contrasts were not performed in patients. The low-dose CT (140 kV, 90 mA, pitch 0.75, 2.5-mm slice thickness, matrix of 512 × 512) was acquired from the skull base to mid-thigh of patients for attenuation correction followed by the whole-body PET scan (3 min per bed position). The PET scans were acquired in 3-D mode in a 512 × 512 matrix and a 2.5 mm slice thickness. Patients lied flat within the body frame on the scanning bed. Marks were made on the body frame and the skin of patients to make sure the repeatability of 2 PET/CT scans. Holding on the scanning position with quiet respiration were required during PET/CT scanning to ensure the quality of images. Images were then reconstructed by attenuation-weighted ordered-subset expectation maximization algorithm (2 iterations, 30 subsets) followed by a postreconstruction smoothing Gaussian filter.

### Image Analysis

PET/CT images were analyzed by a nuclear medicine physician who knew all data of PET/CT scanning, together with an experienced radiation oncologist who knew clinical data. Maximum standardized uptake values (SUV_max_) were calculated automatically and fixed-percentage threshold segmentation method was performed. On the FDG PET/CT images, 50% of the SUV_max_ was applied in segmentation.^18–20^ On the FLT PET/CT images, 50%, 60%, 70%, 80%, and 90% of the SUV_max_ were used. The metabolic volumes (MVs) were automatically calculated by adding up the automatic delineations on the FDG PET images in transverse section slice by slice and named MV50. Similarly, the proliferative volumes (PVs) (PV50, PV60, PV70, PV80, and PV90) were derived from the FLT PET images. Conventional images including contrast-enhanced CT were used to help define boundaries of MV and PV. Mean SUV (SUV_mean_) within MV50 and PV50 were then calculated and labeled as FDG-SUV_mean50_ and FLT-SUV_mean50_.

The CT images from FDG PET/CT scanning was rigidly coregistered to the CT deriving from the FLT PET/CT scan on the same PET/CT scanner automatically, which allowed coregistration of 2 series of PET images. When the automatic coregistration process resulted in significant deviation between the CT series, the 2 CT series were then coregistered manually according to the fixed anatomy adjacent to the tumor, including the vertebral bodies or large vessels. Overlap volume (OV) between MV and PV was delineated manually and calculated by adding up the manual delineations in fusion PET image slice by slice automatically. Overlap ratio (OR) was determined as MV∩PV/PV, where ∩ denotes the intersection, which equals OV. OR50, OR60, OR70, OR80, and OR90 were derived from MV50 overlapping with PV50, PV60, PV70, PV80, and PV90 respectively. All values were calculated on a lesion-by-lesion basis.

### Treatment Planning and Dosimetric Evaluation

The dual series of PET/CT images were loaded into the radiotherapy treatment planning software, Philips Pinnacle^3^ Medical System (Philips Radiation Oncology Systems, Fitchburg, WI). We made 2 IMRT plans for each patient: conventional radiotherapy plan delineated on CT images; and BTV boost planning with a base dose as in conventional plan and boosted dose in subtargets that delineated on PET/CT images. Definitions of conventional gross tumor volume-CT, clinical target volume (CTV)-CT, and planning target volume (PTV)-CT followed the recommendations of the ICRU Report No. 83.^21^ Four-dimensional CT was applied to define internal target volume^22^; 50% of SUV_max_ in FDG PET^18–20^ and optimal threshold in FLT PET based on the above spatial overlap analysis were selected as thresholds for BTV boost in our study. Thus, we defined the regions with union of MV50 and selected PV for biological boost as BTV, and PTV-boost was defined by BTV along with the same individualized margin used in PTV-CT.^22,23^ The following organs at risk (OAR) were contoured: lungs, spinal cord, esophagus, and heart. Parameters and corresponding dose-limiting constraints included the following: mean lung dose ≤20 Gy, lung volume receiving 20 Gy or more (V20) ≤35%, lung volume receiving 5 Gy or more ≤65%; maximal irradiated dose (D_max_) of spinal cord ≤50 Gy; mean esophagus dose ≤ 34 Gy, D_max_ of esophagus ≤105% of prescribed dose; and mean heart dose ≤35 Gy, heart volume receiving 40 Gy or more ≤80%, heart volume receiving 60 Gy or more ≤30%. The dose to the PTV-boost was planned as a simultaneously integrated boost. During the planning optimization, 99% of PTV-boost was required to be covered by the 95% isodose area of the boost and 95% of the PTV-CT to be covered by the 95% isodose area of the conventional radiation.

Both IMRT plans were theoretical and not used for treatment. Tumor control probability (TCP) and normal tissue complication probability (NTCP) were calculated for both treatment plans using the LQ-Poisson-based model^24^ and relative seriality model.^25^ Parameters fitting to the relative seriality model during NTCP determination referred to existed data.^26^

### Statistical Methods

As a pilot study to explore spatial correlation between FDG and FLT PET/CT imaging, at least 20 patients were required to determine initial outcomes. Paired tests comparing the mean values or the medians of the 2 variables surveyed on the same object, either the paired *t* test (for normal distribution) or the paired Wilcoxon signed-rank test (for nonnormal distribution). Results were reported with 95% confidence intervals when available. Statistically significance were set as the 2-sided *P* *<* 0.05. Analysis was performed using SPSS (version 19.0).

## RESULTS

### Characteristics of Patients

Thirty-one NSCLC patients were prospectively included and 23 patients were included for final analysis (Table 1). Patients were excluded from analysis because of single FDG PET/CT imaging (1 patient), poor imaging quality inadequate for analysis (2 patients for malignant pleural effusion and 1 patient for extensive pneumonia), and any anticancer treatment between dual PET/CTs (chemotherapy in 2 patients and radiotherapy in 2 patients).

**TABLE 1:**
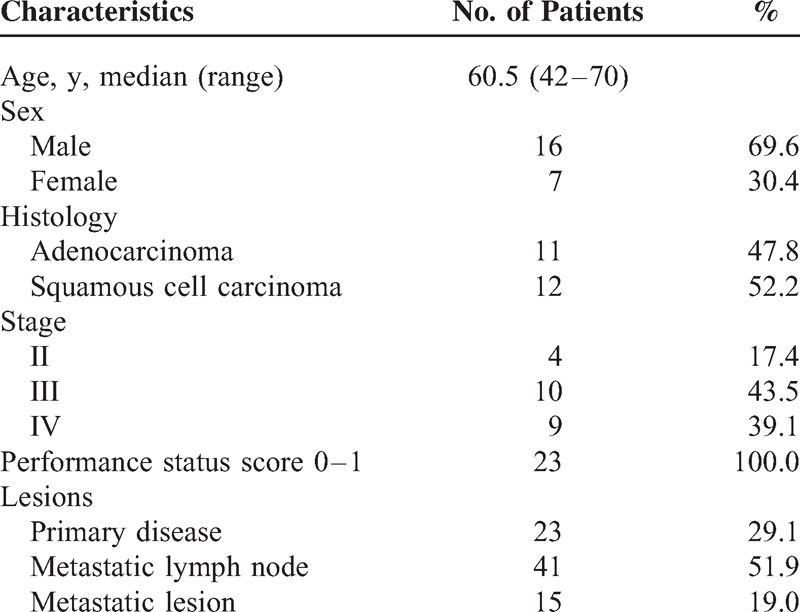
Patient Characteristics

### Spatial Location Relation Between High-Uptake Regions of FDG and FLT PET/CT

**FIGURE 1:**
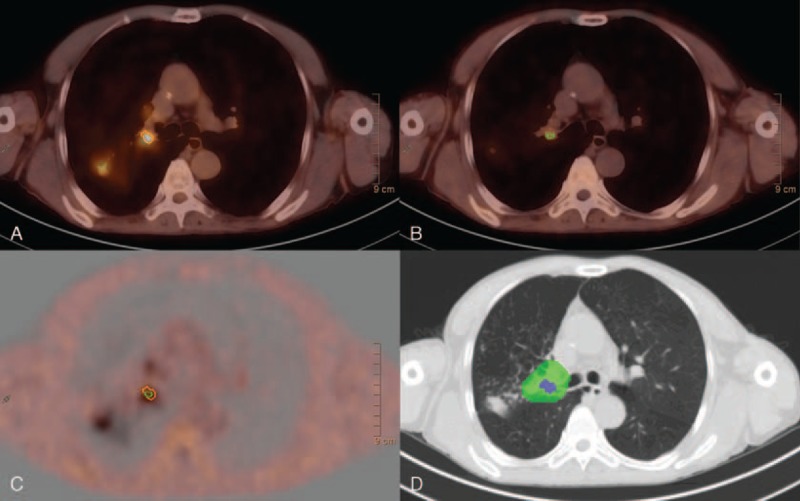
Representative (A) FDG PET/CT, (B) FLT PET/CT, (C) fused PET/CT images, and (D) delineation of target regions of typical primary disease of case 4. The orange lines and the green lines indicate the high-uptake area with threshold of 50% of FDG-SUV_max_ and 80% of FLT-SUV_max_. The green hatched region indicated conventional planning target volume whereas the purple hatched region indicated biological subvolume for boost. CT = computed tomography, FDG = fluorodeoxyglucose, FLT = fluorodeoxythymidine, PET = positron emission tomography, SUV_max_ = maximum standardized uptake value.

According to routine examinations and FDG PET/CT imaging, 85 lesions were determined as positive. FLT showed avidity for 79 of the 85 (92.9%) lung lesions imaged with FDG. All primary tumors (23/23) (4 in upper lobe and 5 in lower lobe of left lung; 5 in upper lobe, 6 in middle lobe, and 3 in inferior lobe of right lung), 89.1% (41/46) of metastatic lymph nodes (5, 3, 2, 4, 2, 5, 2, 3, 6, 5, and 4 lesions in 2R, 3, 4R, 5, 6, 7, 8, 10L, 10R, left supraclavicular, and right supraclavicular regions, respectively) and 93.8% of distant lesions (15/16) (3 abdominal lymph nodes, 3 retroperitoneal lymph nodes, 4 cervical nodes, 1 axillary lymph node, and 4 soft tissue metastases), showed accumulation of dual tracers. For limitation of exhibition of brain metastasis in FDG PET/CT and bone metastasis in FLT PET/CT, distant metastasis including for analysis were mainly nonregional lymphatic and soft tissues metastases. In Figure 1, representative images of typical primary disease are shown.

Tables 2 and 3 summarized main variables deriving from dual tracer PET/CTs. Compared with FDG, SUVs of FLT was significantly lower (*P* *<* 0.001). MV50 were found larger than PV50 (*P* *=* 0.05). Diverse ORs were derived based on different thresholds. Median values increased from OR50 of 58.61% to OR90 of 93.12% as delineating threshold increased.

**TABLE 2:**
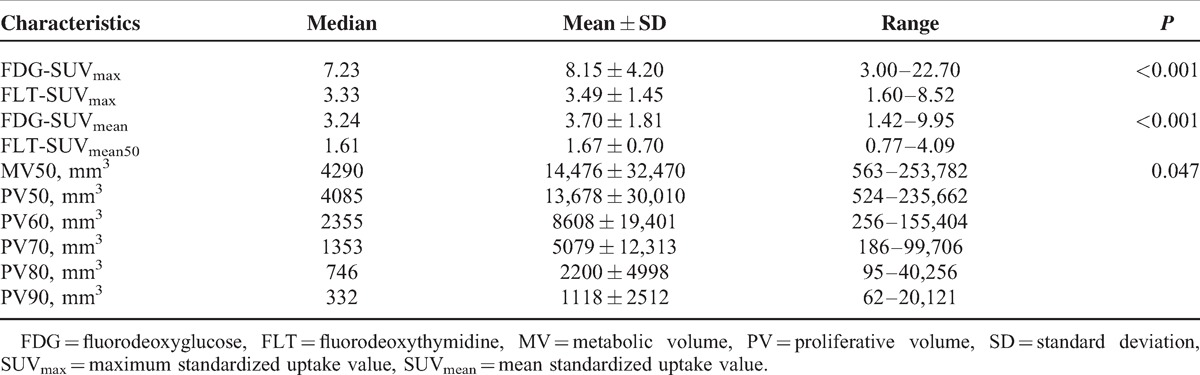
Metabolic Indices and Proliferative Indices of Total Nonsmall Cell Lung Cancer Patients

**TABLE 3:**
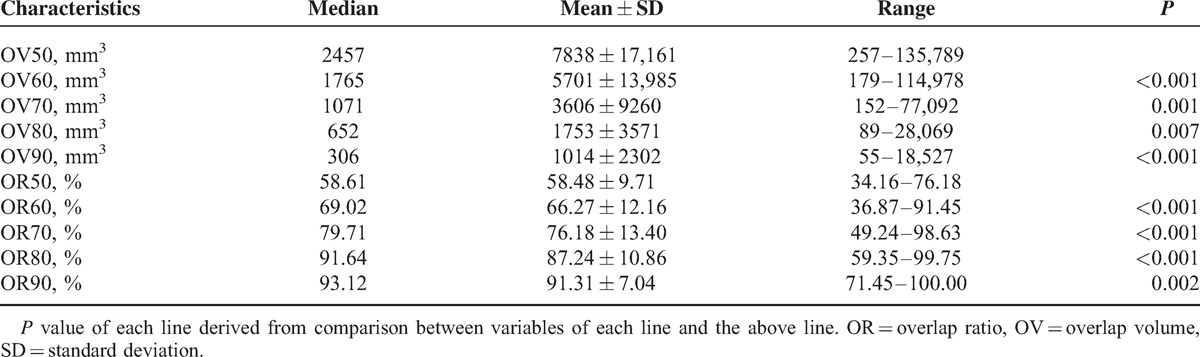
Parameters of Spatial Overlap of Fluorodeoxyglucose and Fluorodeoxythymidine Positron Emission Tomography–Computer Tomography of Total Nonsmall Cell Lung Cancer Patients

### Dosimetric Parameters of IMRT Plans With and Without BTV Boost

**FIGURE 2:**
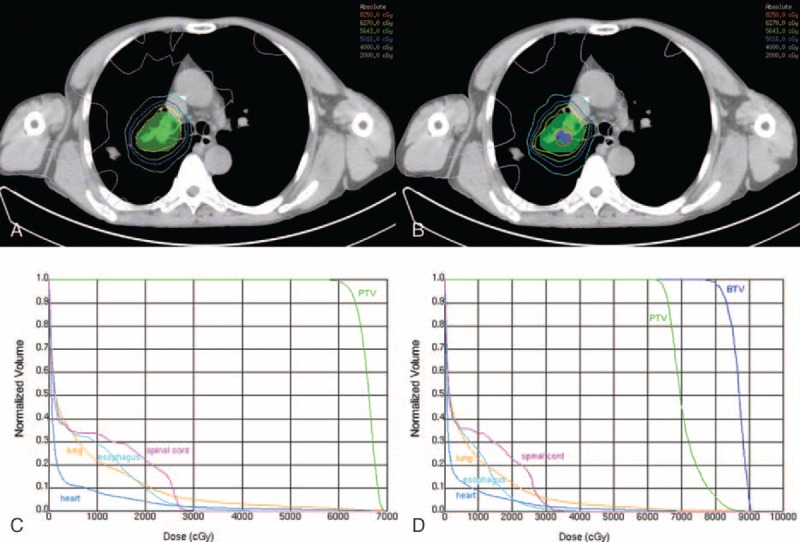
(A and B) Beam configuration and isodose curves and (C and D) dose–volume histograms of conventional and biological boosted intensity-modulated radiation therapy plans for case 4. The green hatched region indicated conventional planning target volume (PTV) whereas the purple hatched region indicated biological subvolume for boost. The red isodose curve is the 82.5 Gy prescribed to boost region, whereas the yellow is 62.7 Gy prescribed to PTV.

The above data showing >90% of PV80 or PV90 were included within MV50. Considering PV90 maybe too small to represent FLT uptake, 80% of SUV_max_ was considered to be optimal for BTV boost. Five NSCLC patients with typical lesions were selected for dosimetric analysis. Table 4 listed tumor characteristics, dose escalation level, TCP, NTCP, and dosimetric parameters derived from dose–volume histogram (DVH) of 5 NSCLC patients. Dose per fractionation was 1.8 to 2.0 Gy for conventional PTV-CT, whereas increased to 2.5 to 2.75 Gy for PTV-boost with simultaneous integrated boost technique. Median total dose were 83.2 Gy for PTV-boost and 60.0 Gy for conventional PTV-CT, respectively. V20 of lung, D_max_ of spinal cord, mean dose of lung, esophagus, and heart derived from DVH were comparable between IMRT plans. The beam configuration, isodose curves, and DVH of case 4 were exhibited as Figure 2. All TCP values calculated were >70%, with higher TCP values of in BTV boost plan in each patient (*P* *=* 0.04). BTV-boost plan resulted in slightly higher NTCP values in comparison with conventional IMRT plans from esophagus, heart, and lung.

**TABLE 4:**
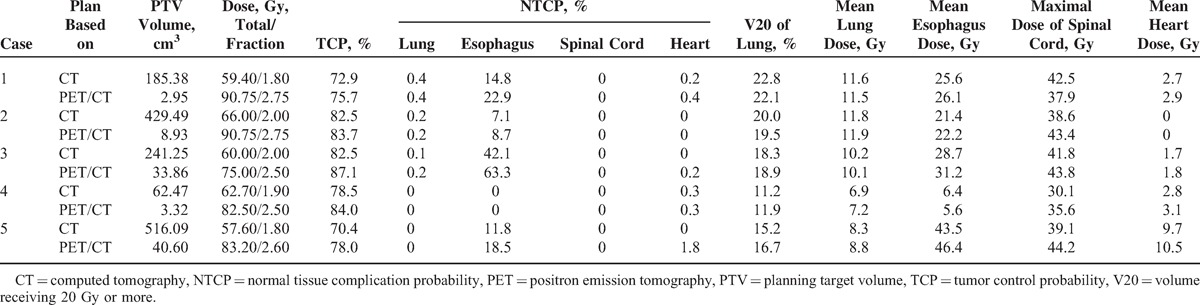
Tumor Characteristic, Dose Escalation Level and Parameters of Dosimetric Evaluation of 5 Nonsmall Cell Lung Cancer Patients for Planning

## DISCUSSION

Interest is growing in radiotherapy to nonuniformly boost radioresistant regions within NSCLC tumors using molecular imaging techniques. The complexity of tumor behavior is beyond the ability of any single radiotracer to reveal. As the only clinically applied radiotracer, specificity of FDG PET-derived tumor volumes was higher than that of CT.^27^ Literatures also showed that NSCLC recurrence was located in strongly FDG avid subvolumes.^28,29^ FLT PET/CT, which has become a research hotspot during recent years, can provide additional biological information of tumor cell proliferation than FDG PET/CT. Therefore, FDG and FLT PET/CT-based definition of target subvolumes may increase the efficacy of radiotherapy by permitting escalated dose to more radioresistant regions. Although most studies focused on quantitative analysis of image parameters derived from FDG and FLT PET/CT, spatial relations between them remain underresearched. In this study, we explored the spatial overlap of glucose metabolism and proliferation of tumor in terms of FDG and FLT imaging, which may help us to define more probable locations of residual cancer and recurrence where should be the target for radiation boost.

Spatial correlations of glucose metabolism and proliferation in terms of FDG and FLT imaging has been explored in few studies.^30,31^ Voxel-based correlation of FDG and FLT PET images were reported to be most highly correlated (*r* *=* 0.76) in oropharynx cancer.^30^ However, FDG was found to display inverse intratumoral distribution patterns with FLT by digital autoradiography analysis of mouse models of human NSCLC.^31^ Here, we found that regions of high FLT uptake being encompassed within the high FDG uptake areas spatially at baseline to a large degree. A threshold of 50% of SUV_max_ was chosen in FDG PET/CT because existed data^28^ showed that MV determined by this threshold overlapped well with the residual disease volume after treatment. In the absence of direct clinical evidence for an association between high FLT uptake regions and a subsequent residual and/or recurrent disease, the influence of pretreatment FLT PET/CT on boost target delineation is still not completely clear. For FLT, uptake was significantly lower than FDG uptake as we shown, a higher threshold than 50% of SUV_max_ used in FDG PET/CT was needed to define specific subregions with high proliferation activity for radiation boost. In existed study,^32^ 80% of SUV_max_ in FLT PET was used as threshold for defining regions with high proliferation activity and performed well for dose escalation. In our analysis, as delineation thresholds in FLT/PET increased, ORs increased significantly from a half to >90%. Delineation <80% of SUV_max_ in FLT/CT resulted in a rather high OR value of 90.79%, indicating that FLT PET/CT provided little information additional to biological subvolume determined by FDG PET/CT.

To further explore the potential effect of spatial relation of baseline FDG and FLT PET on guiding biologically personalized radiotherapy, 2 IMRT plans based on conventional enhanced CT with and without boost of biological subvolumes were created for 5 patients with typical diseases. The radiobiological effects on cancer and healthy cells can be generally characterized by the TCP and NTCP. BTV boost IMRT plans met the prescription goal and OAR constrains as conventional IMRT plans, but provided enhanced therapeutic ratio by increasing TCP without significant increasing NTCP of OARs.

To the best of our knowledge, spatial overlap between high uptake of FDG and FLT and dual PET tracer-guided biological radiation boost for treatment-naive NSCLC patients has not been reported. Delivering a higher dose to the subvolumes with both actively glycolytic and proliferating parts of the tumor at the beginning of the treatment course might have an additive effect and could further reduce the potential of the tumor to recur through accelerated proliferation, repopulation, and other mechanisms. Although the mechanism is not definitely clear, our results showed that the vast majority proportion of high FLT uptake subregions derived from 80% of SUV_max_ threshold located within subregions of high risk for poor local control determined by FDG uptake. The individualized margin added to the delineated BTV to overcome respiratory motion and set-up errors may encompass additional FLT determined subvolume besides of FDG-based BTV. Furthermore, tumor cell repopulation during radiotherapy, which is often inconsistent with baseline proliferation activity both quantitatively and spatially, has been found to be an important indicator of tumor aggressiveness and of its resistance to anticancer treatment. Real-time exploration of tumor cell repopulation that depends on FLT PET has been certified during radiation.^33^ Thus, we may suppose that delivering a dose escalation to FLT PET/CT-guided subvolumes of actively proliferation during radiation might have an additive effect on local control. Note that validation is mandatory before the use of dual tracer PET of FDG and FLT for guiding tumor target delineation is accepted for clinical practice.

However, the results have been mixed, probably for the following reasons. First, spatial overlap analysis and subsequent IMRT comparison in our study were based on the hypothesis that degree and localization FDG and FLT uptake on PET/CT images were rigorously consistent with glucose metabolism and cell proliferation certified by histopathology and immunohistochemistry. Relevant researches are ongoing in our institution. Second, rigorous rigid registration was applied to ensure effectiveness of parameters derived from PET/CT, and thus the convincement of results. Deformable tissue changes were out of consideration in registration. The reproducibility of the deformable registration techniques is limited and it is difficult to validate. Additionally, all IMRT plans in our study were theoretical and were not used for treatment, and patients positioning between PET/CT scans did not completely followed the RT stabilization procedure. Body frame, marks on frame and body surface, and strict positioning procedure ensured the repeatability of scan positioning and accuracy of image coregistration. There is no doubt that radiation stabilization procedure will be applied in the future work.

There is great potential for both FDG and FLT PET/CT applied for biological target volume delineation in radiation dose escalation in NSCLC patients based on our findings, though the clinical outcomes were not considered in this study. FLT PET/CT may be optional during boost subvolumes delineation because of well encompassment of high FLT uptake region within FDG-based subvolume and little information provided for dose escalation and subsequent local control benefit. Based on the current results, we may start a clinical phase I/II study of subregional dose escalation comparing dual PET/CT-guided IMRT and FDG-only-based IMRT for patients with NSCLC.
